# Dynamics of initial drop splashing on a dry smooth surface

**DOI:** 10.1371/journal.pone.0177390

**Published:** 2017-05-11

**Authors:** Zhenlong Wu, Yihua Cao

**Affiliations:** 1 National Laboratory of Aeronautics and Astronautics, Beihang University, Beijing, China; 2 School of Aeronautic Science and Engineering, Beihang University, Beijing, China; Queensland University of Technology, AUSTRALIA

## Abstract

We simulate the onset and evolution of the earliest splashing of an infinite cylindrical liquid drop on a smooth dry solid surface. A tiny splash is observed to be emitted out of the rim of the lamella in the early stage of the impact. We find that the onset time of the splash is primarily dependent on the characteristic timescale, which is defined by the impact velocity as well as the drop radius, with no strong dependence on either the liquid viscosity or surface tension. Three regimes are found to be responsible for different splashing patterns. The outermost ejected droplets keep extending radially at a uniform speed proportional to the impact speed. Finally, we discuss the underlying mechanism which is responsible for the occurrence of the initial drop splash in the study.

## Introduction

The splashing dynamics of a liquid droplet upon a dry solid surface as an important and complicated phenomenon has been accomplished by various scientists during the last decades. This phenomenon plays a crucial role in various disciplines of nature and technological applications [1, 2], e.g., interaction of raindrops with aircraft surfaces in rainfall [3] and icing [4] conditions, spray combustion of liquid fuel [5], ink-jet printing [6], and surface painting and coating [7]. As to our research of interest, i.e., effects of rainfall on aircraft aerodynamics [8, 9], there is an increasing demand to deeply investigate the raindrop splashing dynamics during the interaction process of a raindrop and a wing surface. As a liquid drop hits a solid surface, it often splashes and breaks up into smaller secondary droplets. Splashing, including corona splashing and prompt splashing [10], is a most singular phenomenon in the case of drop impact onto a dry solid surface, which is still not fully understood due to the underlying instability in the breakup phase. Previous studies [10–15] have found that corona splashing owes its existence to the presence of the ambient gas since reducing gas pressure suppresses and even eliminates splashing entirely. These results are motivating new studies on splashing dynamics [16–21].

Recently, Boelens, et al. have studied the pressure effect on splashing of an infinite cylinder on a dry surface [22]. However, more researches are necessary to explain the splashing dynamics and mechanisms for such shaped droplets. Here we examine the onset and evolution of the splashing of an infinite cylindrical liquid drop on a smooth dry solid surface. Since we focus only on the influence of the liquid properties, rather than the air properties, on the dynamics of the initial liquid drop splashing, the properties of the air remain constant. We use a two-dimensional volume-of-fluid (VOF) code [23] to simulate the impact and splash. The VOF methodology has been extensively applied as a robust approach to study drop impact related issues [24–29]. In general, our results show that creation of secondary droplets is observed in the early stages of spreading, and the splashes continuously evolve afterwards, moving forwards at an approximately uniform speed proportional to the impact speed and breaking up into more tertiary droplets.

## VOF model theory

In the present simulation, we include viscosity and surface tension and solve the Navier-Stokes equations with the standard piecewise-linearly interpolated interface between the liquid and the gas phases [23]. Both phases are constrained to incompressible media (S1 Fig). In the VOF model, the *N* phases are considered as one effective fluid throughout the whole domain. Properties such as density *ρ* and viscosity *μ* of this effective fluid are defined as weighted average of each of the *N* phases as follows,

$$\rho =\sum_{i=1}^{N}\alpha_{i}\rho_{i}$$ (1)

$$\mu =\sum_{i=1}^{N}\alpha_{i}\mu_{i}$$ (2)

$$\sum_{i=1}^{N}\alpha_{i}=1$$ (3)

where ${\hat{n}}_{w}$ and ${\hat{t}}_{w}$ are the unit vectors normal and tangential to the wall, respectively.

The tracking of the interface between the phases is accomplished by the solution of a continuity equation for the volume fraction of each phase, which has the following form:

$$\frac{1}{\rho_{i}}\left[\frac{\partial }{\partial t}\left(\alpha_{i}\rho_{i}\right)+\nabla \cdot \left(\alpha_{i}\rho_{i}\vec{U_{i}}\right)=\sum_{j=1}^{N}\left({\dot{m}}_{ji}-{\dot{m}}_{ij}\right)\right]$$ (4)

where $\vec{U_{i}}$ is the velocity vector of phase *i* and ${\dot{m}}_{ij}$ is the mass transfer from phase *i* to phase *j*.

The above volume fraction equation is solved through explicit time discretization with standard finite-difference interpolation schemes applied to the volume fraction values that were computed at the previous time step, i.e.,

$$\frac{\alpha_{i}^{n+1}\rho_{i}^{n+1}-\alpha_{i}^{n}\rho_{i}^{n}}{\Delta t}V+\sum_{f}\left(\rho_{i}U_{f}^{n}\alpha_{i,f}^{n}\right)=\left[\sum_{j=1}^{N}\left({\dot{m}}_{ji}-{\dot{m}}_{ij}\right)\right]V$$ (5)

where *n* and *n*+1 denote indexes for previous time step and current time step, respectively. *α*_*i*,*f*_ is the *i*^*th*^ volume fraction at face *f*, *U*_*f*_ is the volume flux through face *f* and *V* represents volume of the cell.

Momentum balance is solved on a very fine square structured grid and the resulting velocity field is shared among the phases. The momentum equation shown below is dependent on the volume fraction of all phases through the properties *ρ* and *μ*,

$$\frac{\partial }{\partial t}\left(\rho \vec{U}\right)+\nabla \cdot \left(\rho \vec{U}\vec{U}\right)=-\nabla P+\nabla \cdot \left[\mu \left(\nabla \vec{U}+\nabla {\vec{U}}^{T}\right)\right]+\rho \vec{g}+\vec{F}$$ (6)

where $\vec{U}$ is the velocity vector of the effective fluid, *P* is the pressure, $\vec{g}$ is the gravity acceleration and $\vec{F}$ is the body force.

The interface curvature and surface tension adopt the default method in FLUENT, i.e., the interface curvature is calculated using a geometric reconstruction (piecewise-linear) scheme to interpolate near the interface between the phases, as shown in the supplementary S2 Fig. A wall adhesion angle in conjunction with the surface tension model is also adopted in the VOF model. The contact angle that the fluid is assumed to make with the wall is used to adjust the surface normal in cells near the wall. This so-called dynamic boundary condition results in the adjustment of the curvature of the surface near the wall. If *θ*_*w*_ is the contact angle at the wall, then the surface normal at the live cell next to the wall is

$$\hat{n}={\hat{n}}_{w}\cos\theta_{w}+{\hat{t}}_{w}\sin\theta_{w}$$ (7)

where ${\hat{n}}_{w}$ and ${\hat{t}}_{w}$ are the unit vectors normal and tangential to the wall, respectively.

**Fig 1:**
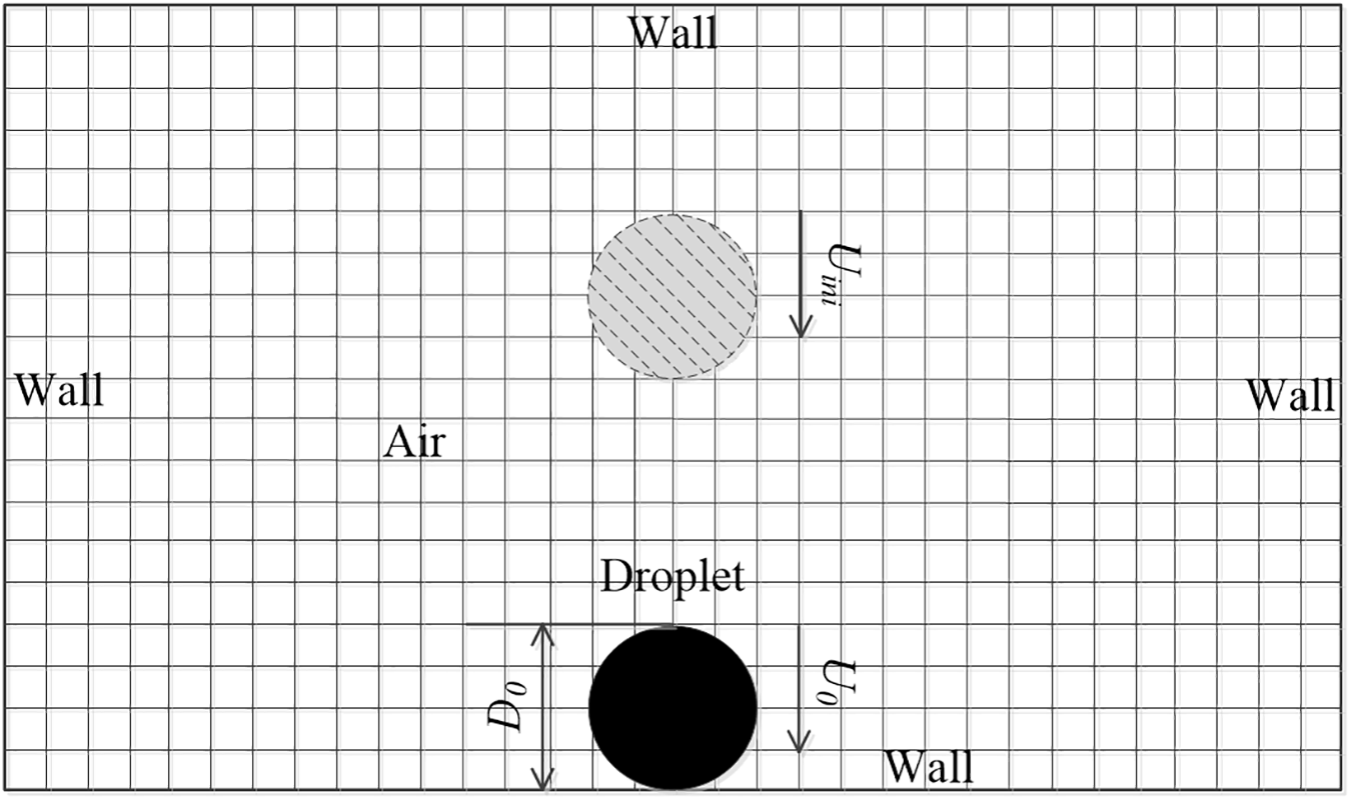
Sketch of the flow domain adopted in this study.

The entire system is enclosed in a rectangular domain which is over 20*a* (*a* represents the radius of the liquid drop) long and 10*a* high, as shown in Fig 1 and the supplementary S3 Fig. A cylindrical droplet is initially falling at a certain height above the substrate with an initial velocity, *U*_*ini*_ (grey shadow in Fig 1) so that the impact velocity equals *U*_*0*_ when contacting the substrate (black solid in Fig 1). The four boundaries of the testing domain are set as no-slip wall. We have carefully checked that the drop splashing behaviors are unaffected by changes in the domain dimensions. Since neither the domain dimensions nor the air properties affect the liquid dynamics reported here, the results rely only on two dimensionless parameters: the Reynolds number *Re* ≡ 2*ρ*_*L*_*U*_*0*_*a/μ*_*L*_ and the Weber number *We* ≡ 2*ρ*_*L*_*U*_*0*_^*2*^*a/σ*, where the symbols will be explained in the later chapters.

## Model accuracy validation

**Fig 2:**
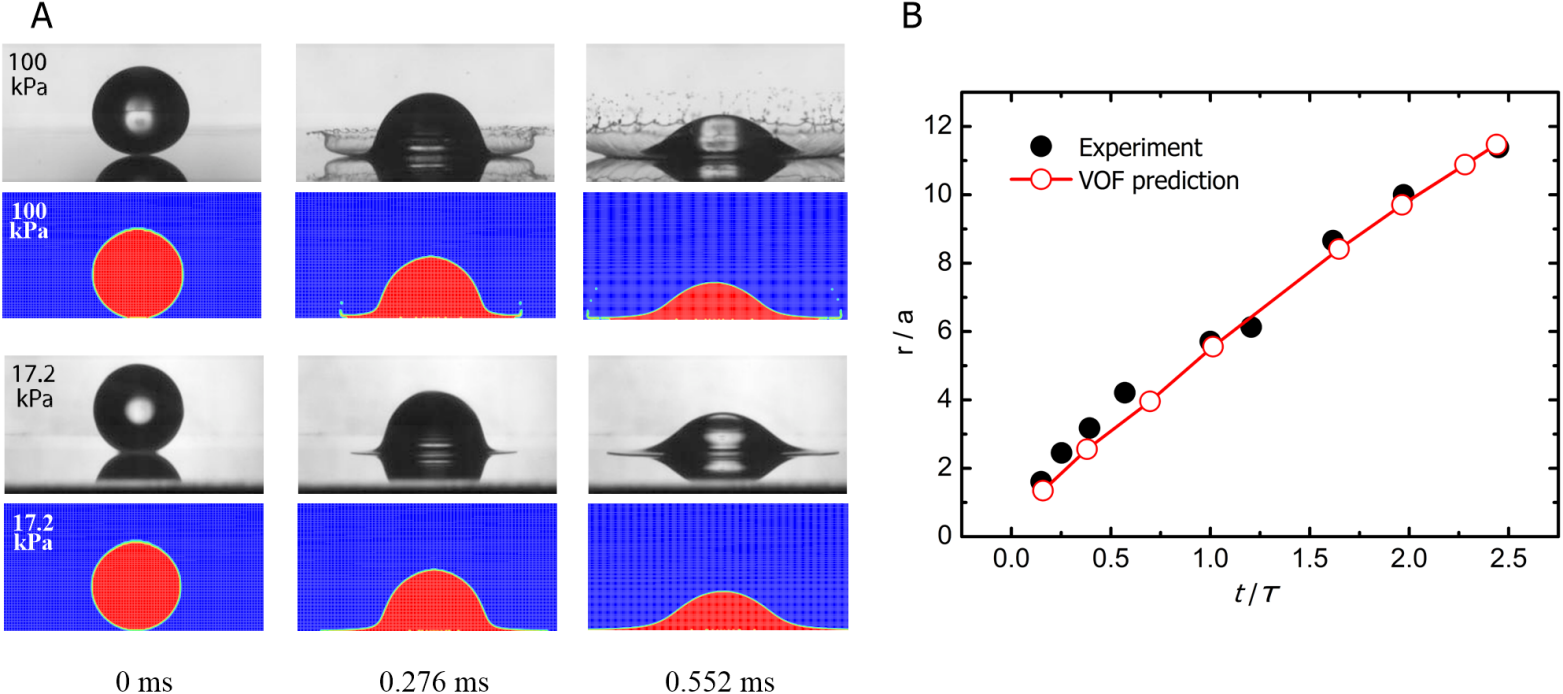
Droplet impact characteristics. A 3.4±0.1 mm diameter ethanol liquid drop impacting a smooth dry substrate at *U*_*0*_ = 3.74±0.02 m/s in the presence of different background air pressures. A. The experimental shapes for a spherical droplet [11] are in black color and the predicted shapes for an infinite cylindrical droplet are in red. In the top and second rows, with the air pressure of 100 kPa, the drop splashes. In the third and fourth rows, under air pressure of 17.2 kPa, there is no splashing at the periphery of the rim. B. Comparison of the radial trajectory of the first ejected droplets impacting a dry solid surface obtained by the experiment and the current VOF prediction.

To validate the present numerical model in the capability of predicting drop splashing, we first calculate the splash at different background air pressures for an ethanol drop hitting a dry solid substrate at an impact velocity *U*_*0*_ = 3.74±0.02 m/s and compare the results for a spherical droplet (colorful contours) experimentally conducted by Xu, et al. [11] and for an infinite cylindrical droplet (monochrome photographs) via the present numerical method, as shown in Fig 2A. The purpose for the comparison between our simulation and the experiment herein is to compare the difference of splashing behavior between the two droplet shapes. It is clearly seen that for both droplet shapes, the air pressure has an identical effect on the splashing characteristics. For the cylindrical droplet at air pressure of 100 kPa, the sheet is pinched off and breaks up into smaller droplets, causing a splash. However, as the air pressure is decreased to 17.2 kPa, the droplet stays attached to the substrate and no splashing occurs. All these phenomena are consistent with what had been observed by Boelens, et al. [22]. Moreover, through comparison, it is found in the same air pressure where splashing occurs, a spherical droplet produces a much more intense splashing than an infinite cylindrical droplet. However, difference also significantly exhibits between the two splashing behaviors. For the spherical droplet, a crown-like structure with lamella detachment is produced. While there is no evident lamella detachment is observed for the infinite cylindrical droplet both here and in the study of Boelens et al [22], which may be attributed to the substantial water surface tension in the longitudinal direction that restrains the formation of lamella detachment. On the other hand, to quantitatively validate the accuracy of the VOF model, we also calculate the splash characteristics of a spherical water drop with radius of 1.7 mm after impacting a dry surface at velocity of 3.8 m/s and compare the results with the experimental data obtained by Stow and Hadfield [30]. To better compare and analyze the outcomes from different impacts with a universal standard, we nondimensionalize the length scales by drop radius *a* and the time scales by *τ*, where *τ* ≡ *a*/*U*_*0*_ is a characteristic falling time for the drop. The normalized radial trajectory of the first (outermost) ejected droplet, *r/a*, with respect to normalized time after first contact, *t/τ*, is plotted in Fig 2B. Quantitatively, our predicted results agree much well with the experimental data and show an approximately linear relationship between the two axial variables.

## Splashing dynamics results

**Fig 3:**
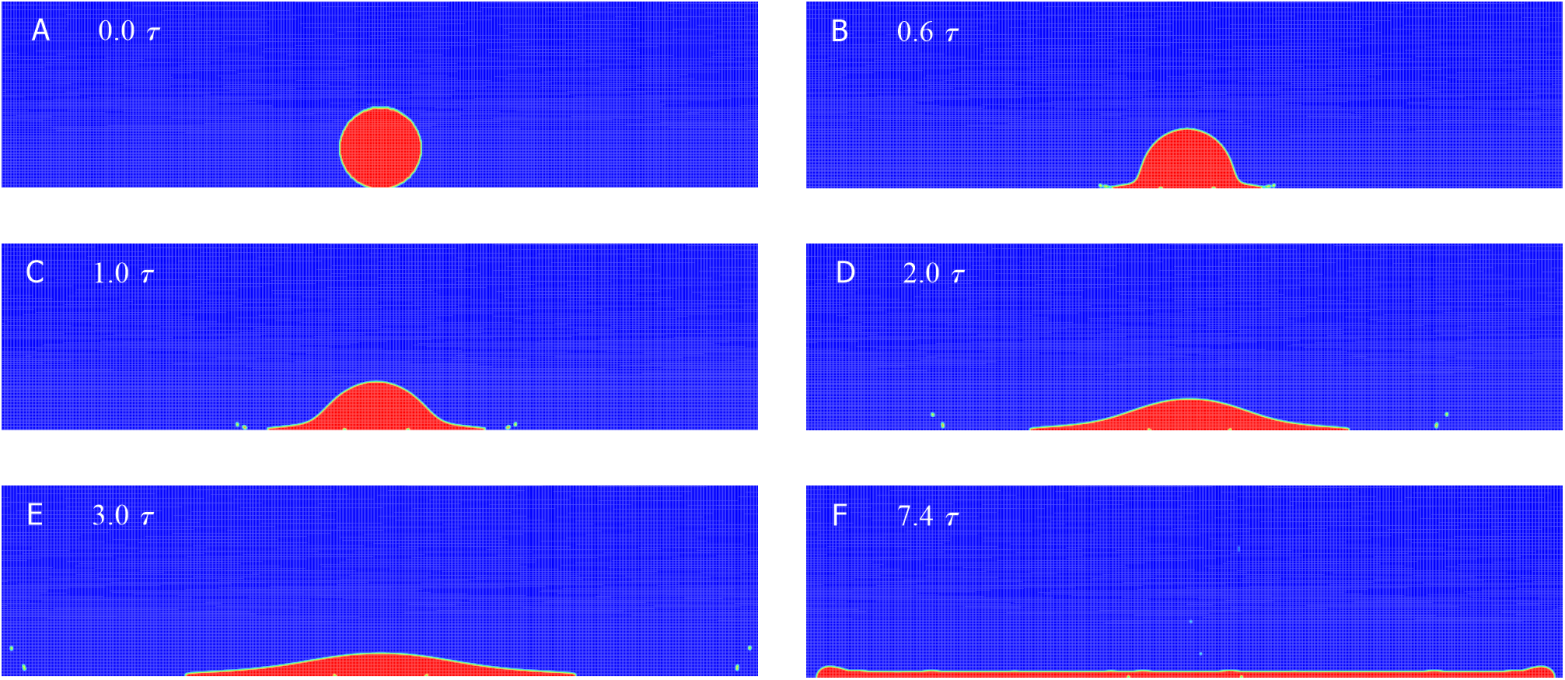
Droplet volume fraction during an impact. Volume fraction contours for a viscous silicone oil drop with diameter of 1.6 mm impacting at 4 m/s onto a smooth dry solid surface under the surrounding pressure of 34 kPa. The successive times are t = 0, 0.6*τ*, *τ*, 2*τ*, 3*τ* and 7.4*τ* where *τ* ≡ *a*/*U*_*0*_ is the characteristic time of impact, as listed in order from A to F.

Fig 3 presents the calculated the splashing behaviors for a liquid silicone oil drop of radius *a* = 1.6 mm contacting a smooth dry substrate at impacting velocity *U*_*0*_ = 4 m/s (a mesh dependence examination is conducted and the results are shown in S4 Fig). The density of the liquid *ρ*_*L*_ = 940 kg/m^3^, as well as the dynamic viscosity *μ*_*L*_ = 9.4 cP and surface tension *σ* = 21 dynes/cm. The gas phase, air, is kept at a pressure of 34 kPa for all simulations in the present study, associated with the density *ρ*_*g*_ = 0.44 kg/m^3^ and dynamic viscosity *μ*_*g*_ = 0.018 cP. Thus, the impact *Re* and *We* correspond approximately to 1280 and 2292, respectively. A static contact angle of 90° is used in all simulations. After contacting the wall [Fig 3A], the drop expands outwards in the radial direction with forward inertia [Fig 3B] and gradually forms a pancake-like lamella with a thickened rim outward [Fig 3C]. Up to *t* = 7.4*τ*, the liquid sheet reaches its maximum radial extent for the first time [Fig 3F]. Meanwhile, we also observed a slight lamella ejecta in the vicinity of the expanding rim at *t* = 0.6*τ* after the drop hits the solid substrate [Fig 3B], causing some tiny secondary droplets propagating much faster than the main lamella. The onset of the splash observed here is well within the regime of the onset of various splashes with the relation of *Oh*(*Re*)^0.609^ = 0.85 or *Oh*(*Re*)^1.25^ = 57.7 empirically fitted by Vander Wal et al. [31] and Mundo et al. [32], respectively, where *Oh* = *We*^1/2^*Re* is the Ohnesorge number. The breakups then extend far away from the rim [Fig 3C to 3E] and finally hit the side walls [Fig 3F].

In the rest of the study, we will examine how the onset of the splashing is controlled by the kinematics of impact, i.e., the liquid properties, the radius and the impacting speed of the drop. Although Xu et al. [11] proposed a relation for the onset of corona splash based on the balance between the restraining pressure of the gas on the spreading liquid and surface tension, expressed by $\sum {}_{G}/\sum {}_{L}=\sqrt{\gamma M_{G}}P\sqrt{\frac{aU_{0}}{2k_{B}T}}\frac{\sqrt{\mu_{L}/\rho_{L}}}{\sigma }$, where $\sum {}_{G}$ and $\sum {}_{L}$ are the destabilizing stress from gas and the stabilizing stress from surface tension, respectively.*γ* is the adiabatic constant of the gas, *M*_*G*_ is the gas molecular weight, *P* is the gas pressure, *T* is the temperature and *k*_*B*_ is the Boltzmann constant. This stress balance shows that the onset of corona splash is essentially affected and can be controlled by the gas pressure. However, it does not tell us when the splashing first emerges. Nor does it depict the temporal and spatial evolution of the splashing structures. Finally, we will present a plot of the spatial patterns and temporal evolution of the splashing and give a comprehensive understanding of the phenomenon.

**Fig 4:**
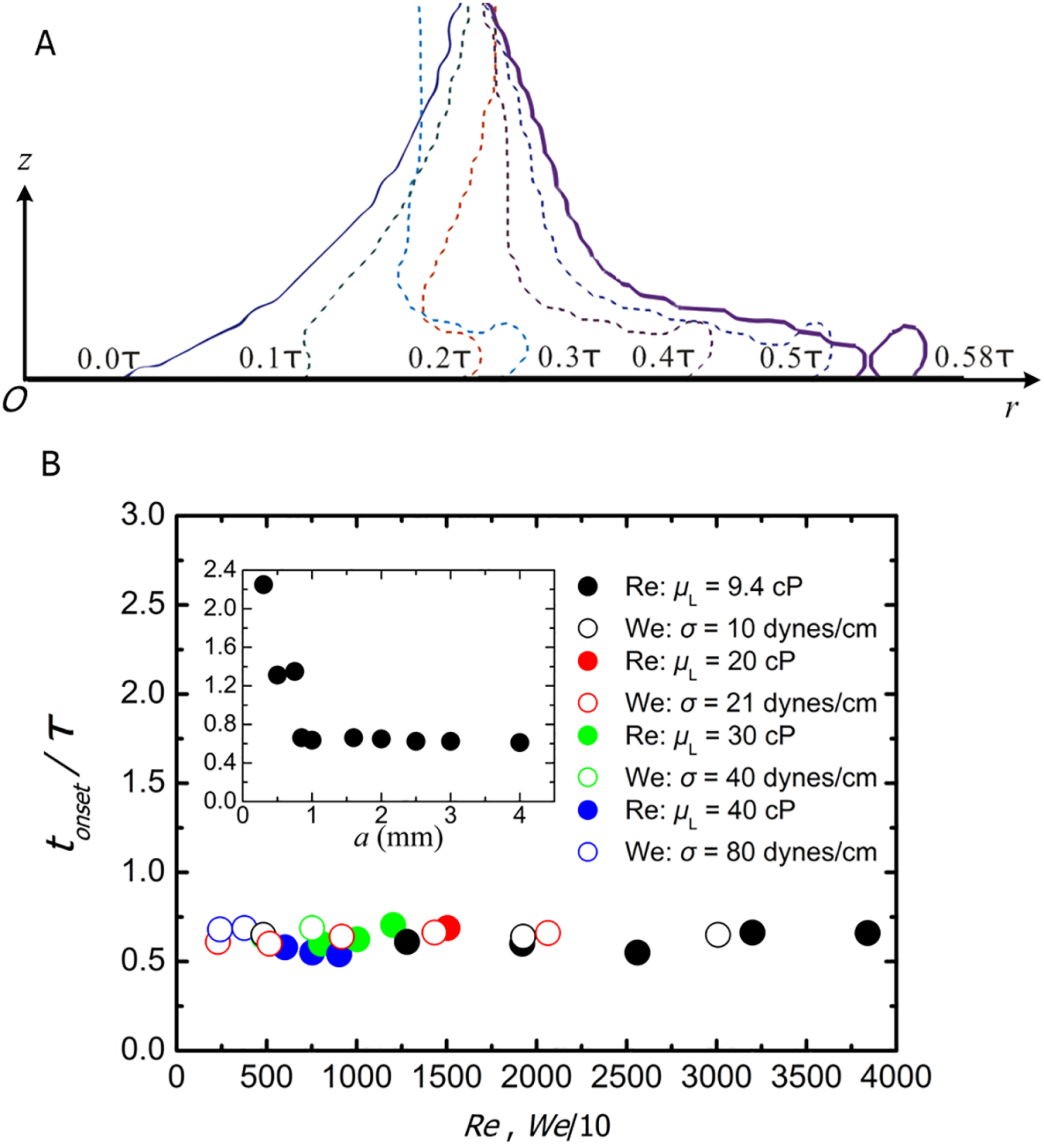
Time evolution of the lamella until splashing emerges. A. Shape evolution for the impact at speed *U*_*0*_ = 8 m/s and the liquid dynamic viscosity *μ*_*L*_ = 40 cP. The other parameters are the same as that for Fig 3. B. Onset time for the splash *t*_*onset*_ as a function of *Re* (solid symbols) and *We* (open symbols), as the drop radius is fixed at *a* = 1.6 mm. To make the two relationships plotted in one figure, the horizontal axis is 10 times reduced for *We*. The different colors correspond to different liquid dynamic viscosity *μ*_*L*_ = 9.4 to 40 cP and surface tension *σ* = 10 to 80 dynes/cm. The inset plots *t*_*onset*_ vs drop radius at the same impact condition as that for Fig 3 except that *U*_*0*_ = 10 m/s.

To track the local evolution of the drop, we adopt a reference frame where the radial axis *r* is along the lamella spreading direction, the vertical axis *z* is along the drop centerline, and the origin *O* is fixed on the substrate [Fig 4A]. We then plot the calculated drop shape profiles within the reference frame from the instant the drop contacts the wall to that the earliest splashing emerges [Fig 4A]. After contact, the no-flux condition at the wall causes the liquid previously falling downward to be diverted into a radially expanding flow. This expanding flow speeds up as it moves away from the centerline. At *t* = 0.2*τ*, a thin collar is found to be ejected from the bottom of the drop. As time goes on, the surface tension slows the edge of the expanding liquid sheet, triggering liquid to accumulate into a round rim. This trend is consistent with the results from previous studies [26, 33, 34]. The rim continues to expand followed by the main part of the lamella spreading slower. By *t* = 0.58*τ*, the outermost rim separates from the lamella in order to balance the excessive energy (*We* = 9168 in this case) which cannot be digested in a single mass merely by drop deformation or viscous dissipation during the drop-wall interactions. Since secondary droplets are emitted out of the rim, for the brevity of description we uniformly treat this rim separation (i.e., lamella breakup) as the onset of splashing of interest in this study, though the breakup is relatively gentle in the early stage of the drop-wall impact.

From the simulation, we can quantify the dynamics by associating the first appearance of splashing with an onset time *t*_*onset*_. Fig 4B plots *t*_*onset*_ nondimensionalized by the characteristic impact time *τ* = *a*/*U*_*0*_ as a function of the drop radius and the impact parameters. From the inset in Fig 4B we can see that the normalized *t*_*onset*_ decreases rapidly with the increasing radius *a* when the drop is relatively small. Within the range of 0.85 to a maximum of 4 mm in this study, the normalized *t*_*onset*_ shows very little change as *a* is increased, whose value is fixed at *t*_*onset*_/*τ* = 0.6 (±0.1). Thus, to reduce the complication of the issue, we fix the drop radius at a = 1.6 mm for the subsequent explorations. The main panel of Fig 4B presents essentially flat curves of *t*_*onset*_ with respect to both *Re* and *We*, which suggests that the dimensional *t*_*onset*_ is predominantly controlled by the characteristic impact time, i.e., the drop size and impact speed, with no strong dependence on either the liquid viscosity or surface tension.

**Fig 5:**
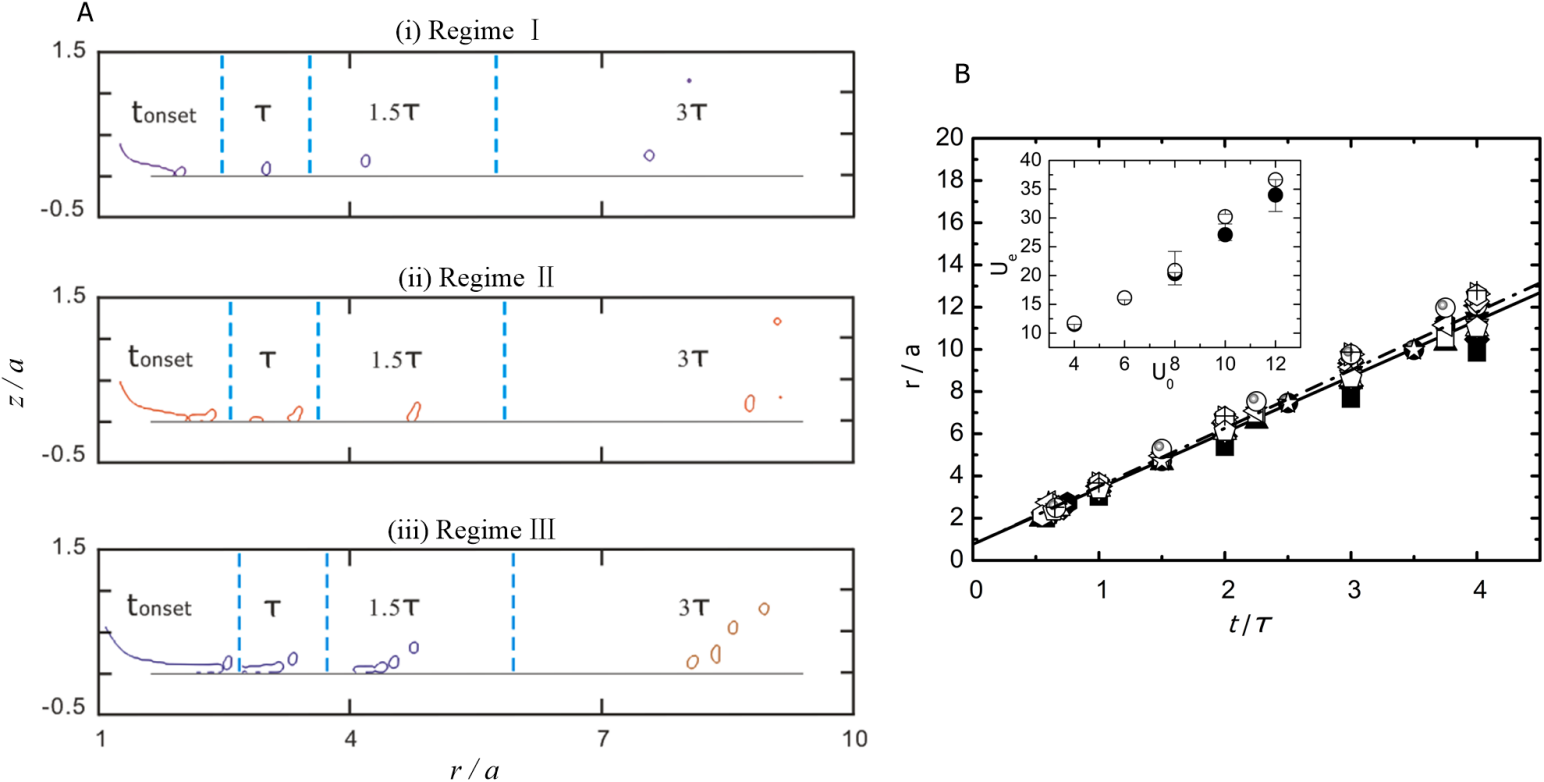
Temporal and spatial evolution of drop splashing in the early stages of impact. A. Three regimes are found to be responsible for the different splashing features shown at the successive times t = *t*_*onset*_, *τ*, 1.5*τ* and 3*τ*. B. Normalized radial distance of the outermost ejected droplet as a function of normalized time at different *Re* (solid symbols, *Re* = 602 to 3840) and *We* (open symbols, *We* = 1354 to 30082). Different symbol shapes denote different values for *Re* or *We*. The inset plots the dimensional radial speed of the outermost ejected droplets *U*_*e*_ vs the drop impact speed *U*_*0*_. The unit for both the speeds is m/s.

Finally, we are going to delineate the features of the splashing from the temporal and spatial evolution of the liquid volume fraction field. Three regimes are observed to be responsible for different splashing patterns, which is closely related to *Re*, as shown in Fig 5A. In RegimeⅠ(*Re* = 602 to 903 in our simulation), an approximately spherical secondary droplet is emitted out of the rim while the remaining portion of the lamella keeps contacting the wall and spreading outwards. In RegimeⅡ(*Re* = 1003 to 1505), a larger irregular liquid parcel is ejected from the lamella. In Regime Ⅲ (*Re* = 1806 to 3840), we see an occurrence of RegimeⅠand RegimeⅡ, accompanied with a spherical secondary droplet ejected out first, while the air is still trapped resulting in a thin air film between the liquid and the wall. After a while, the air destabilizes and breaks up the lamella, causing more secondary droplets moving downstream. For all the three regimes, the ejected droplets extend outwards a very short distance outside the body of the drop and break up into more tertiary droplets.

We also plot the normalized radial distance of the outermost ejected droplet with relationship to the normalized time in the early stages of splashing [Fig 5B]. It is confirmed that for small t/*τ*, the first ejected droplets are not overtaken by droplets released later, so that the vertical axis indicates the trajectory of the first ejected droplets. The incipient splash becomes visible at *t* ≈ 0.6*τ* and r ≈ 2.5*a*, verifying that the normalized displacement is independent of the initial conditions for impact [30]. On the other hand, over the range of time of interest, the displacement of the outermost ejecta shows a straight line with nearly an identical gradient of 2.65 and 2.75 (by fitting the data in Fig 5B) respectively for different *Re* and *We*, implying that the dimensional radial speed of the outermost ejected droplets, *U*_*e*_, has a proportional relationship only to the impact velocity *U*_*0*_, as is apparently indicated in the inset of Fig 5B.

## Splashing mechanism

**Fig 6:**
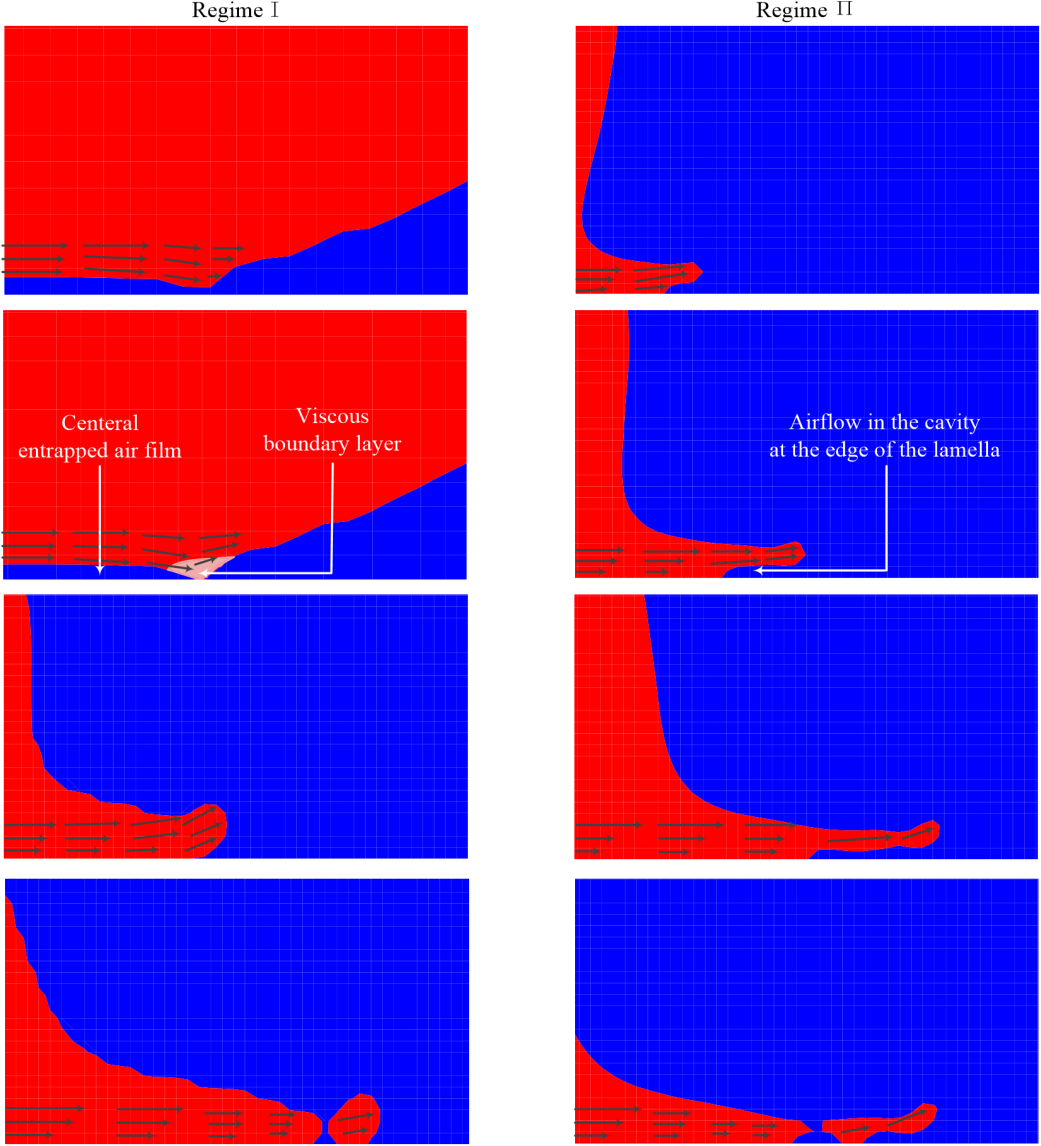
Splashing mechanism. A schematic summarizing the postulated mechanisms for the initial drop splash in RegimeⅠ(left) and RegimeⅡ(right). Shown on the left is a sequence of volume fraction contours starting from the instant the air film ruptures or otherwise allows contact, while on the right is a sequence starting from an instant the lamella shows significant upward deflections but has not been broken up.

In this section, we give our physical understanding of the mechanism for destabilizing the system and causing the occurrence of a splash on a smooth surface. This splashing of RegimeⅠcan be explained by the combining influences of the touchdown of the liquid and the air entrapment between the liquid and the substrate. Once contact occurs, a viscous liquid boundary layer is developed near the contact region [17], as shown in the left-column images in Fig 6. The viscous drag imparts the horizontal flow of the liquid an abrupt resistance and decelerates the liquid in contact with the substrate. To conserve the total flux of volume, the horizontal flow must be diverted away from the surface, i.e., the viscous boundary layer obtains a vertical velocity component normal to the substrate. The normal velocity directs the individual fluid parcels away from the wall. As time goes on, the diverted flow enters the new formed lamella, causing the lamella to take off from the wall and form a splash. As *Re* is increased to be within RegimeⅡ, due to the relatively large horizontal velocity plotted in the inset of Fig 5B, the lamella moves more rapidly in the horizontal direction than in the vertical direction. On the other hand, a cavity emerges at the edge of the spreading drop in RegimeⅡ, as shown in the right-column images in Fig 6. The airflow in the cavity “cushions” the impact and destabilizes the lamella [13], producing a bulk of lamella breakup rather than the regular spherical liquid parcel in RegimeⅠ. The combined action of the above two factors in Regime Ⅰ and Regime Ⅱ causes the splashing pattern in Regime Ⅲ. Finally, it should be noted that though the central air film is always present upon the initial contact between the drop and the substrate, we found no significant air film beneath the spreading drop at the time of sheet ejection in our simulation as well as in other experiments [13]. Therefore, the splashing mechanism in Regime is attributed to the air flow at the edge of the spreading drop rather than the central air film, which is consistent with previous splash experiments [11, 13].

## Conclusion

In conclusion, we have primarily studied the onset and evolution of the corona splashing of an infinite cylindrical liquid drop impacting a smooth dry solid surface and found that the onset time for the earliest splashing is primarily dependent on the impact time, i.e., the drop radius and impact speed, while the radial speed of the outermost ejected droplet is uniform at all times, whose value is only proportional to the impact speed. The flow deflection in the viscous liquid boundary layer and the air entrapped between the liquid and the substrate can explain the occurrence of the splashing. Our results are of vital importance in studying the behavior of supercooled large droplet (SLD) impinging on airfoils in aviation meteorology and other areas.

## Supporting information

S1 Fig Air velocity contours at the instants of interest for drop impact speed of 12 m/s. (DOC)

S2 Fig Interface calculation approach adopted in Fluent VOF model. (DOC)

S3 Fig Mesh topology in the present study. (DOC)

S4 Fig Mesh independence examination results. (DOC)
