# Supplementary material for: Dynamics of initial drop splashing on a dry smooth surface
Source: PLoS One. 2017 May 11;12(5):e0177390. doi: 10.1371/journal.pone.0177390 (PMC5426750; doi:10.1371/journal.pone.0177390)
Supplement: S1 Fig — (DOC) [file pone.0177390.s001.doc]

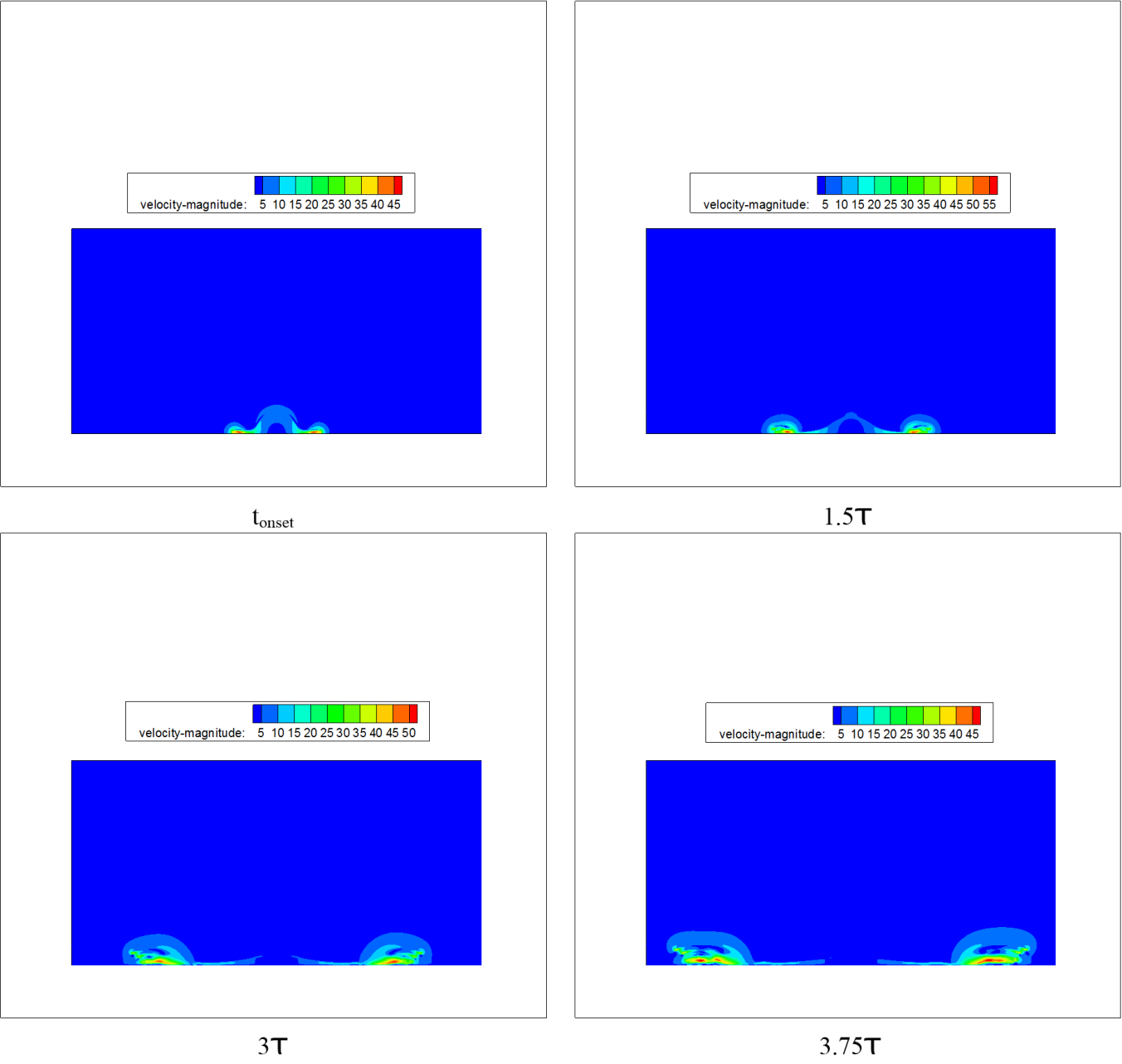


**S1 Fig. Air velocity contours at the instants of interest for drop impact speed of 12 m/s.**

In regard to the air compressibility, we give a plot of the air velocity contours starting from the onset time of splashing at impact speed of 12 m/s, the maximum impact velocity in our study, as shown in S1 Fig. It is found that the maximum air velocity is near 55 m/s, which corresponds to Mach number (Ma) of 0.16. As is known in the field of aerodynamics, air compressibility needs to be considered when Ma>0.3. Thus, it is reasonable to adopt the impressible air model in the present study.
