# Supplementary material for: Dynamics of initial drop splashing on a dry smooth surface
Source: PLoS One. 2017 May 11;12(5):e0177390. doi: 10.1371/journal.pone.0177390 (PMC5426750; doi:10.1371/journal.pone.0177390)
Supplement: S2 Fig — (DOC) [file pone.0177390.s002.doc]

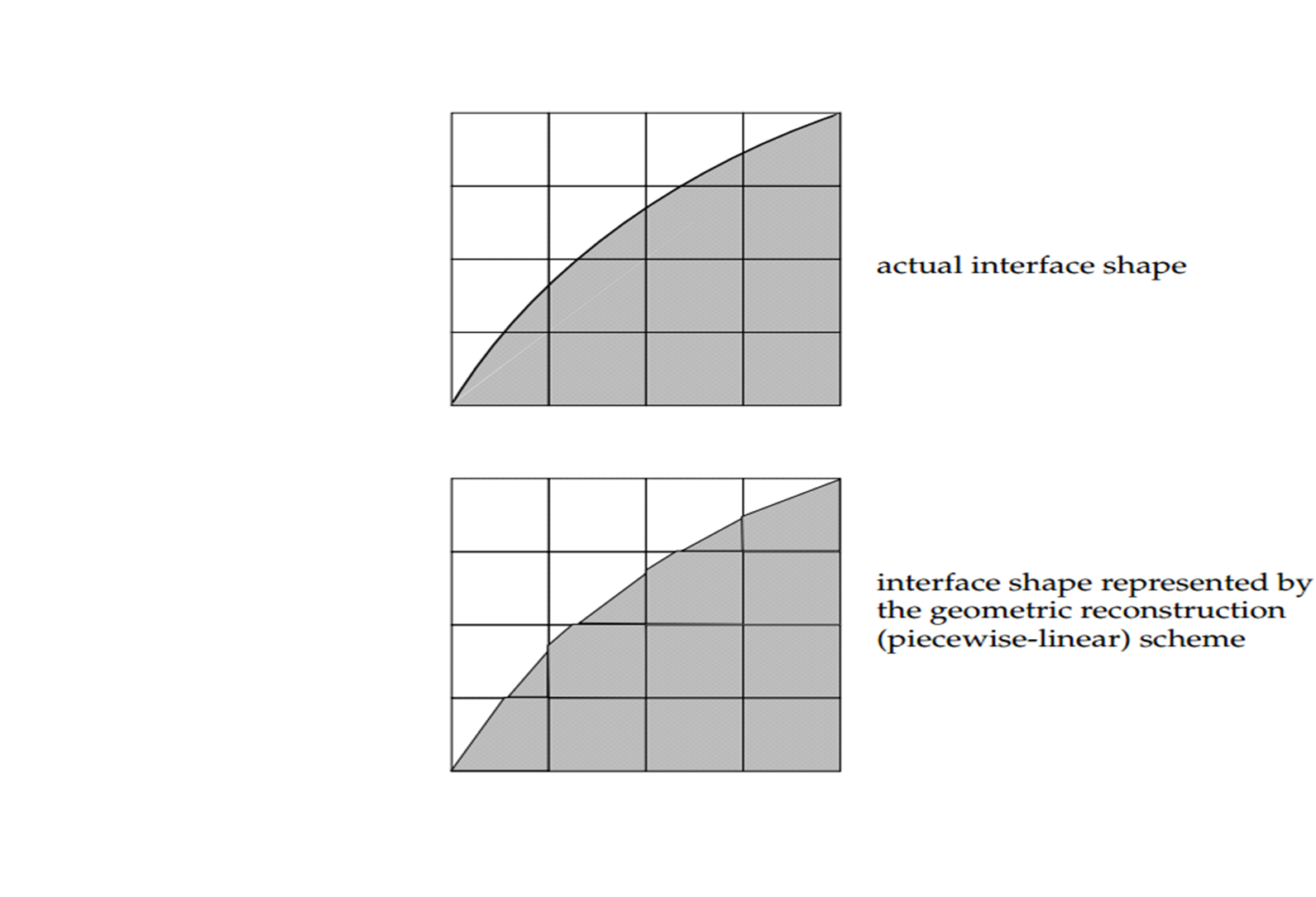


**S2 Fig. Interface calculation approach adopted in Fluent VOF model.**

The interface curvature and surface tension adopt the default method in FLUENT, as has been made clear in the revised manuscript. The interface curvature is calculated using a geometric reconstruction (piecewise-linear) scheme to interpolate near the interface between the phases, as shown in the above S2 Fig.
