# Supplementary material for: Dynamics of initial drop splashing on a dry smooth surface
Source: PLoS One. 2017 May 11;12(5):e0177390. doi: 10.1371/journal.pone.0177390 (PMC5426750; doi:10.1371/journal.pone.0177390)
Supplement: S3 Fig — (DOC) [file pone.0177390.s003.doc]

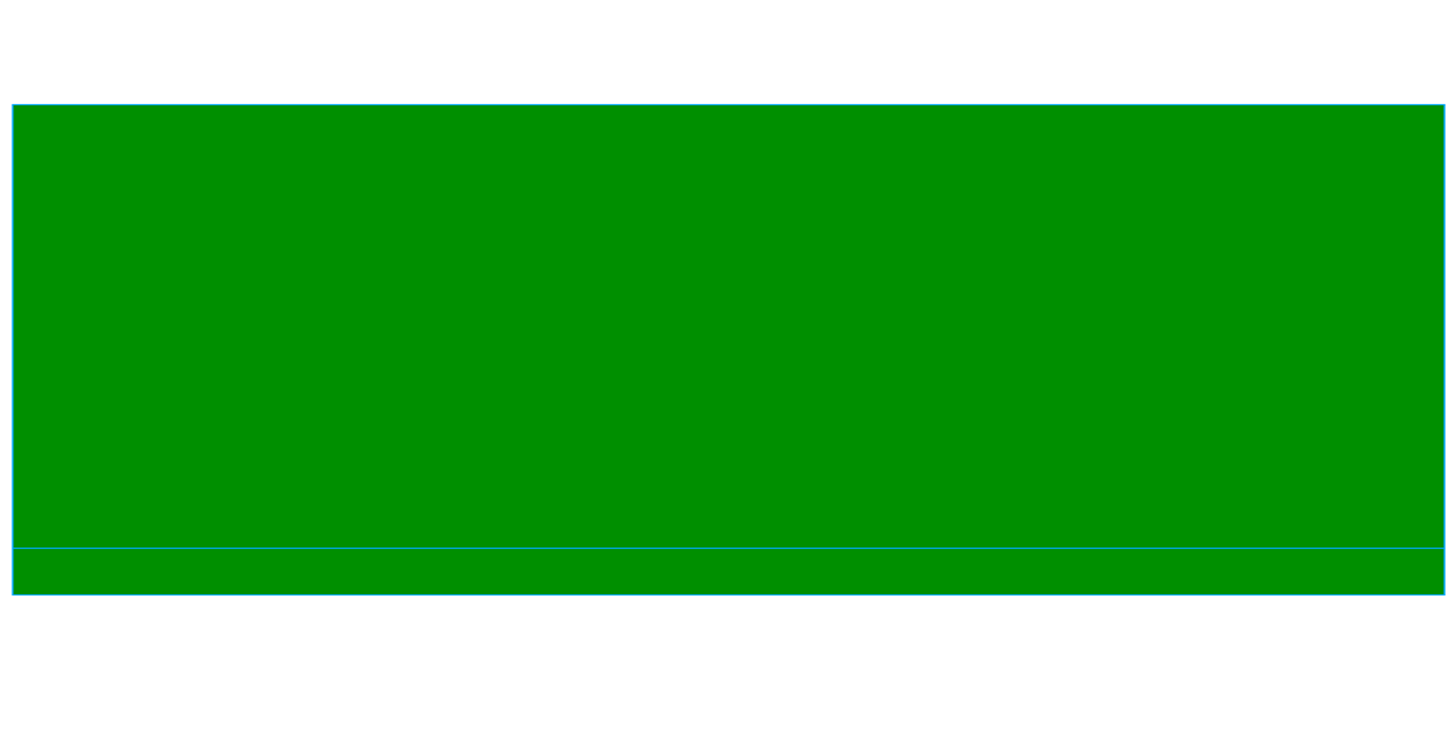


**S3 Fig. Mesh topology in the present study.**

In the present simulation, the rectangular computational domain is of 0.042 m long and 0.021 m high. A lower subgrid (below the green highlighted line in the domain) is established where the drop impacts and interacts with the wall which allows for denser grid point distributions in the vertical direction, as well as an upper subgrid with sparse grid point distributions. The lower subgrid has 100000 and 100 grid points uniformly distributed horizontally and vertically, respectively and the upper one has 200 progressively sparse grid points in the vertical direction. The near-wall mesh gives a smallest grid size of 420 nm. From a computational point of view, this is the smallest feasible mesh size that we can adopt for this system. In order to achieve complete calculation convergence, we set the temporal resolution as high as 0.1 μs. In practice, using these spatial and temporal resolutions, the necessary physics of splashing are already present and we expect the main observations of our simulations to be qualitatively correct. The liquid drop is traced through this grid plane via the VOF model (technical details can be referred to Fluent Tutorials).
