# Supplementary material for: Dynamics of initial drop splashing on a dry smooth surface
Source: PLoS One. 2017 May 11;12(5):e0177390. doi: 10.1371/journal.pone.0177390 (PMC5426750; doi:10.1371/journal.pone.0177390)
Supplement: S4 Fig — (DOC) [file pone.0177390.s004.doc]

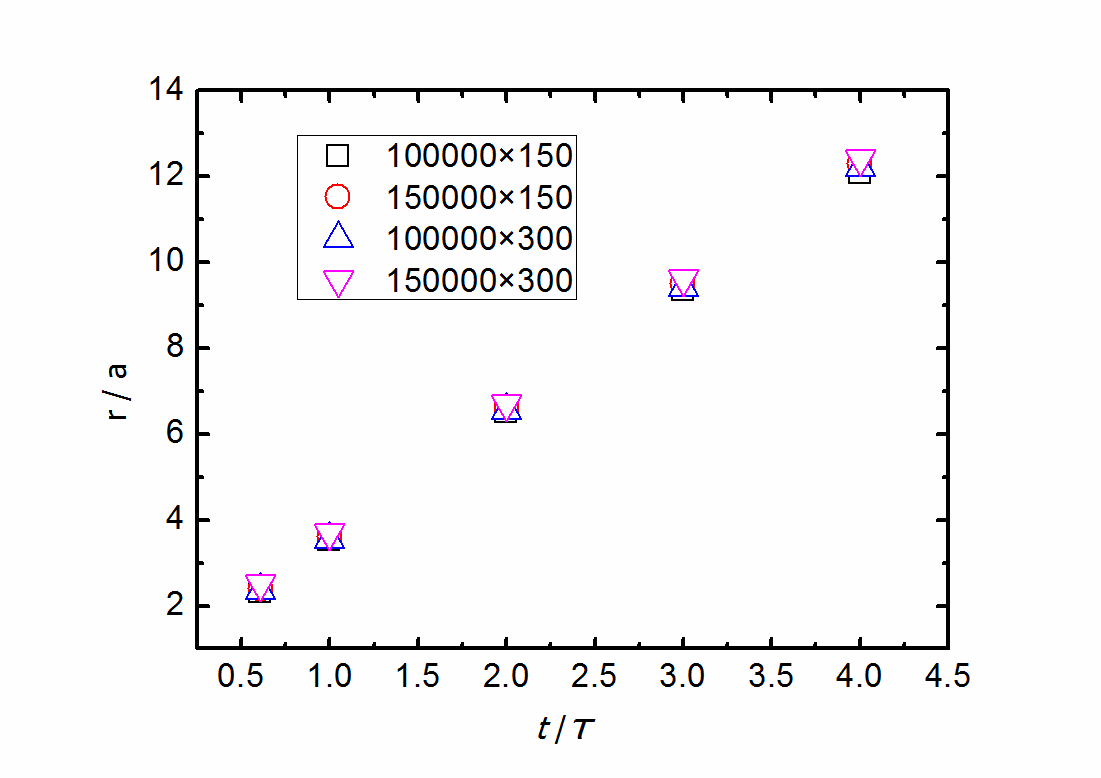


**S4 Fig. Mesh independence examination results.**

Normalized radial distance of the outermost ejected droplet as a function of normalized time for four-level mesh refinement. (*a* = 1.6 mm, *U_0_* = 4 m/s, *Re* = 1280, *We* = 2292). The above results obtained for different grid dimensions reveal no significant mesh discretization influence on the accuracy of the results.
